# Post-mortem cardiac-specific troponin-I (cTnI) to determine the cause of non-traumatic hemopericardium

**DOI:** 10.1007/s12024-025-01043-2

**Published:** 2025-07-10

**Authors:** Hans H. de Boer, Ethan D. Sutton, Maria Pricone, Sarah Parsons

**Affiliations:** 1 Dept. of Pathology, Victorian Institute of Forensic Medicine, 65 Kavanagh Street, Southbank, VIC 3006 Australia; 2 Dept. of Forensic Medicine, Monash University, 65 Kavanagh Street, Southbank, VIC 3006 Australia; 3 Bachelor of Biomedical Science Honours, School of Public Health and Preventive Medicine, Monash University, 553 St Kilda Road, Melbourne, VIC 3004 Australia; 4 Dept. of Toxicology, Victorian Institute of Forensic Medicine, 65 Kavanagh Street, Southbank, VIC 3006 Australia

**Keywords:** Troponin, cTnI, Hemopericardium, Aortic dissection, Myocardial infarction, Autopsy, Pathology, Sudden cardiac death, Biochemistry, Forensic medicine

## Abstract

Hemopericardium is regularly seen at autopsy and post-mortem imaging. Once traumatic cases and resuscitation artefact are excluded, hemopericardium is almost always due to either ruptured myocardial infarction or aortic dissection. In this study, we explored whether post-mortem cardiac-specific troponin I (cTnI) can be helpful when autopsy is not feasible and post-mortem imaging findings are inconclusive. Post-mortem cTnI levels were compared between 46 cases with hemopericardium due to ruptured myocardial infarction (RMI), 38 cases of hemopericardium due to aortic dissection (AoD), and 44 cases of natural deaths without hemopericardium (controls). The results showed significantly higher cTnI levels in the RMI group (median 5,821 ng/L) compared to AoD (median 273 ng/L) and controls (median 95 ng/L). Receiver operating characteristic (ROC) analysis indicated that a cTnI threshold of 1,483 ng/L provided the best balance of sensitivity (87%) and specificity (90%) for distinguishing RMI from AoD. Levels of cTnI above 9688 ng/L were exclusively seen in RMI, whilst levels < 250 ng/L excluded this diagnosis. Calculated likelihood ratios demonstrated that higher levels of cTnI favour RMI over AoD, but substantial overlap between the cohorts limited the diagnostic value of intermediate cTnI values. Autopsy remains the gold standard for determining the cause of hemopericardium and cTnI testing is best reserved for cases in which an autopsy is not feasible.

## Highlights

The diagnostic utility of cardiac Troponin I (cTnI) to determine the cause of non-traumatic hemopericardium was studied.

Post-mortem cTnI serum levels were overall much higher after myocardial rupture than aortic dissection.

A cTnI treshold of 1,483 ng/L provided optimal sensitivity and specificity.

Levels > 9,688 ng/L excluded aortic dissection, levels < 250 ng/L excluded myocardial rupture.

Intermediate cTnI levels were diagnostically inconclusive.

## Introduction

Cardiac-specific troponin (cTn), which includes cardiac troponin I (cTnI) and cardiac troponin T (cTnT), is a protein complex integral to the regulation of cardiac muscle contraction. Elevated serum levels of cTnI and cTnT are well-established markers for myocardial injury and cTn testing is one of the cornerstones of the clinical diagnosis of acute myocardial infarction [1–3].

Multiple studies have explored the diagnostic utility of cardiac-specific troponin in post-mortem settings. These studies generally indicate that elevated post-mortem troponin levels correlate with ante mortem myocardial damage [4–7]. However, the interpretation of post-mortem concentrations remains challenging due to several key limitations. Firstly, any myocardial injury – regardless of aetiology – results in troponin release, meaning elevated levels are non-specific and cannot, in isolation, indicate a particular cardiac pathology. This limitation is further complicated by variability in mode and timing of death. For instance, in individuals who experience a sudden rapid death, troponin levels may not yet be elevated, whilst those undergoing a prolonged dying process may show raised levels due to secondary myocardial injury rather than a primary cardiac cause of death. Research has also shown that post-mortem cTn levels may not remain stable over time, further undermining their diagnostic reliability [8]. As a result, the diagnostic value of post-mortem cTn testing remains uncertain.

The increased use of post-mortem CT (PMCT) scanning in autopsy practice presents a novel context in which post-mortem troponin testing might be useful. PMCT imaging is now widely used to screen and triage cases, guiding decisions on whether an internal examination is warranted, and to help determine its scope and extent. A frequent PMCT finding is hemopericardium. Once ante mortem trauma is excluded as cause, hemopericardium is typically due to one of three aetiologies: a ventricular rupture following myocardial infarction, aortic dissection (AoD), or as an artefact of perimortem cardiopulmonary resuscitation (CPR).

Autopsy easily and reliably differentiates between these causes and offers the added advantage of detailed macroscopic and histological evaluation, which can help detect underlying pathology, including genetic aortic or cardiac diseases [9]. However, autopsy is not always feasible due to legal, logistical, financial, religious or ethical constraints.

Certain PMCT features may assist in distinguishing between ruptured myocardial infarction, aortic dissection or CPR-artefact. For example, crescentic hyperdensity within the hemopericardium – the so-called ‘armoured’ or ‘hammered’ heart – is considered a vital sign that effectively rules out CPR-related artefact [10–12]. Conversely, the presence of a fluid-fluid level in the hemopericardium is considered by some to be suggestive of a CPR-artefact [12, 13]. Aortic dissection may be indicated by findings such as intimal tears, luminal calcifications, or differential sedimentation within the true and false lumina [14]. A ruptured myocardial infarction may be suggested by heterogeneous attenuation of the myocardial wall or epicardial fat [15]. These features may however be subjective, inconclusive and not universally present. PMCT angiography can enhance diagnostic certainty in ambiguous cases, but requires specialized training, equipment and extended procedural time and is not always available.

In cases where an autopsy is not performed and radiological findings are inconclusive, post-mortem cTn testing may help clarify the cause of the hemopericardium. Since ruptured myocardial infarction requires significant myocardial necrosis, substantially elevated serum cTn levels would be expected. In contrast, deaths due to hemopericardium following aortic dissection are assumedly mostly rapid, with presumably limited myocardial injury and consequently lower cTn concentrations. If this holds true, post-mortem cTn serum levels may help differentiate between hemopericardium due to ruptured myocardial infarction and that resulting from aortic dissection. The research in this paper aims to evaluate this hypothesis.

## Materials and methods

This study was conducted at the Victorian Institute of Forensic Medicine (VIFM); a statutory authority responsible for medically investigating all deaths reportable under the Victorian Coroners Act [16]. Approximately, 7500 deaths are referred to VIFM annually, comprising a wide range of natural, unnatural, and unexpected deaths.

A total of 128 cases were included in the research. These included 46 cases of hemopericardium due to a ruptured myocardial infarction (RMI) and 38 cases of hemopericardium due to aortic dissection (AoD). Unfortunately, no cases of artefactual, CPR-related hemopericardium were identified during the study period. To establish a post-mortem baseline of cTnI, we therefore also included 44 random cases of natural death without hemopericardium (controls). All cases showed no visible signs of decomposition.

Each case underwent full body PMCT followed by an autopsy with histological examination of all major organs. Post-mortem cardiac troponin I (cTnI) testing was also performed. For this, femoral vein blood samples were collected immediately upon admission to the institute. To minimise post-mortem biochemical alterations, the blood samples were centrifuged at the earliest reasonable opportunity and the resulting serum was immediately stored at -20 °C.

Troponin testing was outsourced to the Royal Melbourne Hospital. For this, the serum sample was defrosted and a small amount was transferred to a smaller tube, refrozen and dispatched. The test was conducted on the same day the samples were received, using the ARCHITECT STAT High-Sensitivity Troponin-I assay (Abbott Ireland Diagnostic Division, Longford, Ireland). This chemiluminescent microparticle immunoassay, preformed on an Abbott Architect i2000 analyser [17], reports cTnI concentrations in nanograms per Litre (ng/L), with a reportable range of 0–50,000 ng/L.

For each case, the cTnI level (in ng/L) and cause of hemopericardium or cause of death were recorded. Group comparisons were made between the RMI, AoD and control groups using a Kruskal-Wallis one-way ANOVA followed by an uncorrected Dunn’s multiple comparisons test. A p-value of < 0.001 was considered statistically significant.

To evaluate the ability of cTnI concentrations to distinguish between hemopericardium due to RMI and due to AoD, a Receiver Operator Characteristic (ROC) curve was generated. In addition, likelihood ratios (LRs) were calculated. In general, LRs expresses the degree that a given test result alters the probability between two competing and mutually exclusive hypotheses. The LR for several ranges of cTnI was calculated by dividing the probability of RMI by the probability of AoD in that particular cTnI range.

## Results

**Fig. 1:**
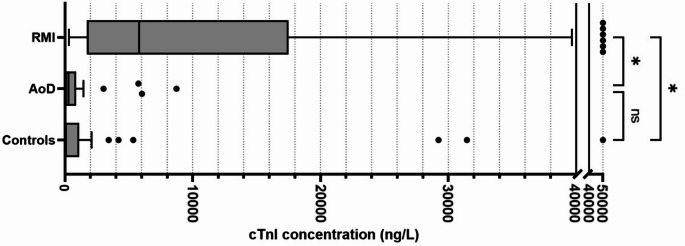
Post-mortem cardiac troponin I (cTnI) concentrations per study cohort. *Boxplot of cTnI concentrations for each group. An uncorrected Dunn’s test was used to assess differences in cTnI levels across cohorts*,* with results indicated (ns = non-significant*, * = p value < 0.0001)

Cardiac cTnI concentrations differed markedly across the three study cohorts. As shown in Table 1, the median cTnI level was highest in the RMI cohort (5,821 ng/L), substantially exceeding that of the AoD cohort (273 ng/L) and the control cohort (95 ng/L). These trends are also visually represented in the boxplot in Fig. 1, demonstrating the notably higher distribution of cTnI concentrations in RMI cases compared to the other groups. Despite a clear difference in central tendency, there was some overlap in cTnI concentrations between the cohorts. At the upper end of the cTnI range, this overlap included four outliers from the AoD group and six outliers from the control group. The RMI cohort showed no outliers at the lower end of the distribution. The five RMI cases with the lowest cTnI concentration all had levels below 1,000 ng/L. There were another eight RMI cases with a cTnI level between 1,000 and 2,000 ng/L.

**Table 1:** Mean, median and range of cTnI serum concentrations per study cohort

		cTnI concentration (ng/L)		
Cohort	**N**	**Mean**	**Median**	**Range**
Ruptured myocardial infarction	46	13,966	5,821	304–50,000
Aortic dissection	38	955	273	3–8,735
Controls	44	3,110	95	3–50,000

*Abbreviations: cTnI = cardiac Troponin I; ng/L = nanogram per litre*

The Kruskal-Wallis one-way ANOVA confirmed that cTnI concentrations differed significantly between the cohorts (x2 (df = 2, *n* = 128) = 55.990, p-value < 0.0001). Post-hoc pairwise comparisons using the uncorrected Dunn’s T-test also revealed significant differences between the RMI group and both the AoD and control groups (p-value < 0.001 for both). No statistically significant difference was found between the AoD and control cohorts (p-value 0.833).

**Fig. 2:**
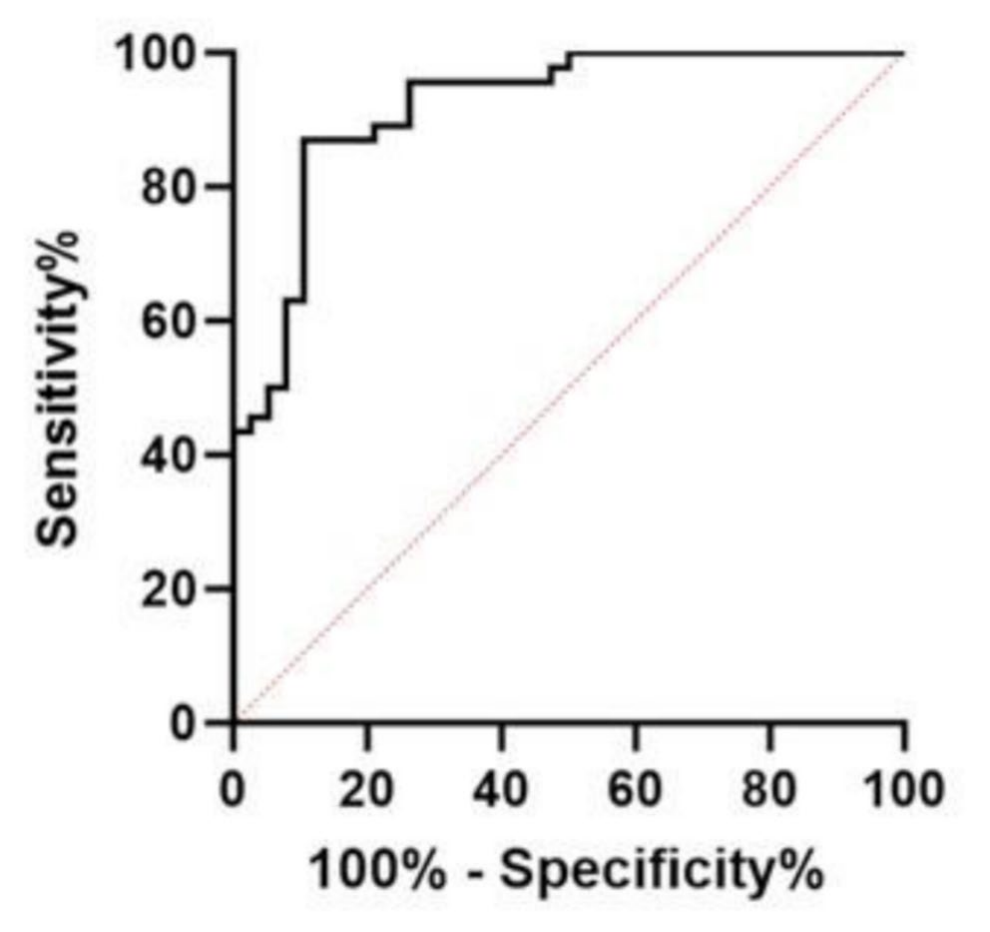
Receiver Operator Characteristic Curve. *Receiver Operating Characteristic (ROC) curve (black line)*,* showing the performance of post-mortem cardiac-specific Troponin I (cTnI) in differentiating between hemopericardium due to ruptured myocardial infarction (RMI) from one due to aortic dissection (AoD). The area under the curve is 0.9188*,* reflecting strong discriminatory capacity*

The Receiver Operator Characteristic (ROC) analysis (Fig. 2) resulted in an area under the curve of 0.9188 (95% confidence interval 0.8601–0.9774) indicating very good diagnostic performance when considering RMI vs. AoD (see also Fig. 2). A threshold of > 1,483 ng/L provided the best combined sensitivity (87%; 95% confidence interval 74-94%) and specificity (90%; 95% confidence interval 76-96%). A cut-off value of > 9688 ng/L was associated with a 100% specificity, meaning that all cases with such a value in our cohort were RMI.

The likelihood ratios (LRs) for specific cTnI ranges are provided in Table 2. These LRs show the effect of a particular test result on the probability of RMI vs. AoD. For example, a cTnI level between 1,501 and 5,500 ng/L has an LR of 11, indicating that such a test result increases the pre-test probability of RMI over AoD elevenfold.

The calculated LRs suggest that, based on our cohort, cTnI concentrations below 250 ng/L exclude RMI, whilst concentrations over 10,000 ng/L exclude AoD as the cause of the hemopericardium. In the intermediate ranges, the diagnostic utility of cTnI varied. Generally, higher cTnI levels were associated with higher LR values, meaning stronger evidence for RMI over AoD. However, exceptions to this trend were noted in the 501-1,000 ng/L and 5,501 − 10,000 ng/L ranges. In both those ranges, the discriminatory power of cTnI was greatly reduced by an almost equal number of RMI and AoD cases. The number of cases in almost all the intermediate ranges was low, limiting statistical power.

**Table 2:** Likelihood ratios for ruptured myocardial infarction vs. aortic dissection

cTnI (ng/L)	RMI (*n* = 46) *N* (%)	AoD (*n* = 38) *N* (%)	LR^1^
1–100	0 (0)	13 (34)	0.00
101–250	0 (0)	6 (16)	0.00
251–500	2 (4)	7 (18)	0.22
501–1,000	3 (7)	3 (8)	0.88
1,001–1,500	1 (2)	5 (13)	0.15
1,501–5,500	15 (33)	1 (3)	11
5,501–10,000	5 (11)	3 (8)	1.38
> 10,000	20 (43)	0 (0)	∞

*Abbreviations: cTnI = cardiac-specific Troponin I; ng/L = nanogram per Litre; RMI = ruptured myocardial infarction; AoD = aortic dissection; LR = likelihood ratio*

^*1*^*Likelihood ratios were calculated by dividing the proportion of RMI cases in each cTnI range by the proportion of AoD cases. For example*,* for the 501-1*,*000 ng/L range*,* 7% / 8% = 0.88*

## Discussion

This study explored the diagnostic utility of post-mortem cTnI concentrations to help determine the cause of non-traumatic hemopericardium, specifically to help distinguish between hemopericardium due to ruptured myocardial infarction (RMI) and aortic dissection (AoD). The results demonstrated that post-mortem cTnI levels are significantly higher in cases of RMI compared to both AoD and controls without hemopericardium, suggesting that elevated cTnI may assist as a biochemical marker of myocardial rupture. Our ROC analysis indicated that a threshold of > 1,483 ng/L achieved the best balance between sensitivity (87%) and specificity (90%). Concentrations above 9,688 ng/L were exclusively associated with RMI.

There is no literature available on typical cTnI levels for ruptured myocardial infarction or aortic dissection, but our findings are plausible from a pathophysiological perspective. The mechanism of myocardial rupture implies substantial pre-existing necrosis of cardiac muscle and thus a substantial release of cTnI prior to death. In contrast, death following hemopericardium due to AoD would not directly damage the myocardium in most cases, which explains the generally lower cTnI levels in that group. The overall tendency in the data supports these assumptions, but there was notable overlap in cTnI levels between the cohorts and there were multiple outliers.

A substantial number of cases of RMI presented with low levels of cTnI, which suggests that not all such cases are associated with large areas of established myocardial necrosis. There were five cases with cTnI level below 1,000 ng/L, and another eight cases with a cTnI level between 1,000 and 2,000 ng/L.

In the AoD cohort, four cases had relatively high cTnI levels. A more detailed analysis of circumstantial information and autopsy findings indicated that all these cases had substantial underlying cardiac disease, and autopsy findings or circumstantial information suggestive of more protracted disease:

- A case with a cTnI level of 6,038 ng/L had subacute dissection of the aorta, severe cardiac hypertrophy (720 g) and severe coronary artery disease. The circumstantial information indicated three days of epigastric pain prior to death.
- A case with a cTnI level of 3,028 ng/L had subacute dissection of the aorta, severe cardiac hypertrophy (718 g) and mild coronary artery disease. There was a history of several days of ‘flu-like symptoms’ prior to death.
- A case with a cTnI level of 8,735 ng/L, had acute-on-chronic aortic dissection, severe cardiac hypertrophy (727 g), moderate coronary artery disease, and focal myocarditis.
- A case with a cTnI level of 5,751 ng/L had acute dissection of the aorta at autopsy but had also been diagnosed with a ST-elevated myocardial infarction 3 days prior to death.

These cases highlight that considerable heterogeneity exists in a cohort of fatal hemopericardium due to aortic dissection. Although a sudden death is most common, a subset appears to present with protracted disease and/or substantial concomitant cardiac disease, which may explain substantially elevated post-mortem cTnI levels. In none of the above four cases the aortic dissection involved the coronary arteries, but this might also explain elevated troponins in other cases of aortic dissection.

In the control cohort, seven outliers had a relatively high cTnI. Some cTnI variability in this group was expected, since these cases were randomly selected from all natural deaths submitted to our institute. In four of these cases, the elevated cTnI level was explained by acute myocardial infarction in the days before death. Another case died in hospital with congestive cardiac failure complicating known dilated cardiomyopathy, and the elevated cTnI was also unsurprising. The last two cases had no significant cardiac pathology, and the cause of death remained unascertained after autopsy. There was therefore no clear cause for the elevated level of cTnI.

The overlap in cTnI concentrations between the cohorts, including the outliers discussed above, had a notable effect on the diagnostic power of cTnI, as especially demonstrated by the calculated LR values. Based on our study, intermediate levels of cTnI are essentially inconclusive, only providing weak support for either AoD or RMI. The LR values suggested that cTnI levels at the lower and higher ends of the spectrum are much more informative, with an LR of zero for cTnI levels below 250 ng/L (effectively excluding RMI), and an infinitively large LR for levels above 10,000 ng/L (effectively excluding AoD).

Several limitations of our study must be acknowledged. One of the primary constraints was the availability of suitable cases. This limited sample size and affected statistical power. Future studies are therefore needed to confirm our results.

Since no cases of CPR-related (artefactual) hemopericardium were encountered during the study period, consideration was focused on the differential diagnosis of ruptured myocardial infarction versus aortic dissection. The study results are therefore most applicable to cases in which artefactual hemopericardium is already excluded by the presence of an ‘armoured’ or ‘hammered heart’ on PMCT, as mentioned in the introduction.

We were also unable to include other (very rare) causes of non-traumatic hemopericardium, such as endocarditis, mycotic aortic aneurysms, or myocardial metastases [18, 19]. It is expected that at least some of these cases are associated with myocardial injury and therefore an elevated post-mortem troponin level. This could lower the likelihood of RMI when elevated cTn levels are encountered. The low pre-test probability of these rare alternative causes of hemopericardium should however be noted.

Some minor technical limitations include restricting our study to cTnI and using a single troponin assay. Clinical literature however indicates that cTnT levels and cTnI are strongly correlated [2, 20] and this is likely transferrable to a post-mortem setting. Also, validated assays are by definition reliable and precise, which mitigates the need to explore technical measurement errors. Our study only relied on easily obtainable femoral vein blood samples and results may differ for other collection sites.

Every effort was made to limit post-mortem alterations of cTnI levels. The samples were taken as early as possible and processed in such a way to minimise degradation. However, our study was performed using the normal operational workflow within our institute, and some variability between the cases in terms of post-mortem interval and technical processing could therefore not be avoided. It cannot be excluded that this affected some of our results. At the same time, this approach ensured that our results are reflective of, and applicable to, our daily casework.

## Conclusion

Post-mortem cardiac troponin I (cTnI) testing can potentially help determine whether a hemopericardium is likely to be caused by ruptured myocardial infarction or aortic dissection. Especially low (< 250 ng/L) or high (> 10,000 ng/L) cTnI serum levels have strong differentiating power. Results in intermediate ranges are inconclusive, although certain values may favour one diagnosis over the other. In general, cTnI testing results should be regarded indicative, rather than diagnostic. An autopsy remains the preferred method to determine the underlying cause of a hemopericardium and cTnI testing should be reserved for cases in which an autopsy is not feasible.

## Data Availability

The data that support the findings of this study are available from the corresponding author upon reasonable request.

## Declarations

### Generative AI and AI-assisted technologies in the writing process

The authors used ChatGPT 3–5 to help improve the readability and language of the manuscript. The authors reviewed and edited any suggestions as needed and take full responsibility for the content of the published article.
